# Supplementary material for: Role and mechanism of NCAPD3 in promoting malignant behaviors in gastric cancer
Source: Front Pharmacol. 2024 Apr 22;15:1341039. doi: 10.3389/fphar.2024.1341039 (PMC11070777; doi:10.3389/fphar.2024.1341039)
Supplement: Supplementary file 11 [file DataSheet2.ZIP › GSEA/immunologic/my_analysis.Gsea.1599462003817/GSE3982_DC_VS_MAC_DN.html]

Details for gene set GSE3982\_DC\_VS\_MAC\_DN[GSEA]

|  || Dataset | filtered\_dataset.sample\_info.cls#WT\_versus\_NCAPD3\_MUT |
| Phenotype | sample\_info.cls#WT\_versus\_NCAPD3\_MUT |
| Upregulated in class | WT |
| GeneSet | GSE3982\_DC\_VS\_MAC\_DN |
| Enrichment Score (ES) | 0.51282 |
| Normalized Enrichment Score (NES) | 1.9081621 |
| Nominal p-value | 0.007352941 |
| FDR q-value | 0.53410935 |
| FWER p-Value | 1.0 |
Table: GSEA Results Summary

  

Fig 1: Enrichment plot: GSE3982\_DC\_VS\_MAC\_DN      
 Profile of the Running ES Score & Positions of GeneSet Members on the Rank Ordered List

  

| SYMBOL | TITLE | RANK IN GENE LIST | RANK METRIC SCORE | RUNNING ES | CORE ENRICHMENT || 1 | 4288 | MKI67 | 1 | 1.341 | 0.1378 | Yes |
| 2 | 55149 | MTPAP | 15 | 1.051 | 0.2371 | Yes |
| 3 | 7170 | TPM3 | 24 | 1.018 | 0.3365 | Yes |
| 4 | 3954 | LETM1 | 47 | 0.924 | 0.4162 | Yes |
| 5 | 5087 | PBX1 | 54 | 0.908 | 0.5057 | Yes |
| 6 | 22879 | MON1B | 151 | 0.735 | 0.5128 | Yes |
| 7 | 51388 | NIP7 | 593 | 0.428 | 0.2411 | No |
| 8 | 57493 | HEG1 | 683 | 0.386 | 0.2173 | No |
| 9 | 6241 | RRM2 | 755 | 0.346 | 0.2021 | No |
| 10 | 8848 | TSC22D1 | 899 | -0.324 | 0.1331 | No |
| 11 | 9388 | LIPG | 937 | -0.361 | 0.1439 | No |
| 12 | 6273 | S100A2 | 942 | -0.365 | 0.1787 | No |
| 13 | 55148 | UBR7 | 987 | -0.396 | 0.1882 | No |
| 14 | 9962 | SLC23A2 | 1169 | -0.537 | 0.1139 | No |
| 15 | 10001 | MED6 | 1190 | -0.561 | 0.1576 | No |
Table: GSEA details [plain text format]

  

Fig 2: GSE3982\_DC\_VS\_MAC\_DN      
 Blue-Pink O' Gram in the Space of the Analyzed GeneSet

  

Fig 3: GSE3982\_DC\_VS\_MAC\_DN: Random ES distribution      
 Gene set null distribution of ES for **GSE3982\_DC\_VS\_MAC\_DN**
